# Combination therapy: synergism among three plant extracts against selected pathogens

**DOI:** 10.1186/s13104-023-06354-7

**Published:** 2023-05-20

**Authors:** Martin Ntiamoah Donkor, Addai-Mensah Donkor, Richard Mosobil

**Affiliations:** 1 Department of Biochemistry and Forensic Sciences, School of Chemical and Biochemical Sciences, C. K. Tedam University of Technology and Applied Sciences, Navrongo, Ghana; 2 Department of Pharmaceutics, School of Pharmacy and Pharmaceutical Sciences, University for Development Studies, Tamale, Ghana; 3 Department of Applied Chemistry, School of Chemical and Biochemical Sciences, C. K. Tedam University of Technology and Applied Sciences, Navrongo, Ghana

**Keywords:** Fractional inhibitory concentration, Synergy, Extract-extract combination, Antimicrobial activity

## Abstract

**Objective:**

The synergism among extracts of *Senna alata, Ricinus communis,* and *Lannea barteri,* and their anti-infective activities were investigated. The data collected for the antimicrobial activity of the extracts combinations were interpreted to be one of the following categories: synergy; indifferent; additive; or antagonistic. The interpretation was made based on the fractional inhibitory concentration index (FICI) results. FICI of ≤ 0.5 indicates synergism, > 0.5 to 1 indicates additive effects, > 1 to ≤ 4 indifference, and > 4 is considered to be antagonism.

**Results:**

Compared with the data of the individual extracts, the MIC values of the extract-extract combinations against all strains of the tested microorganisms were significantly lower, ranging from 0.97 to 1.17, 0.97 to 4.69, 0.50 to 1.17, 1.17 to 3.12 and 2.34 to 4.69 mg/mL for *Escherichia coli, Staphylococcus aureus, Pseudomonas aeruginosa, Klebsiella pneumonia and Candida albicans* respectively. *L. bateri* aqueous-*S. alata* ethanol extracts and *S. alata* aqueous-*R. cummunis* ethanol extracts combinations showed a synergy effect against all the test microorganisms. The other combinations exhibited at least one additive effect. Neither antagonism nor indifference activity was observed. This study validates the relevance of combining these plants in treating infections by traditional medicine practitioners.

**Supplementary Information:**

The online version contains supplementary material available at 10.1186/s13104-023-06354-7.

## Introduction

Advances in the treatment and prevention of disease rely on novel therapeutics, which are based on innovative drug targets. Scientists dedicate a significant amount of time and money to exploring the new potential targets essential to continuing medical advancements [1].

Antibiotics have become increasingly less active against certain ailments not only because they create noxious responses but also due to the upsurge of drug-resistant microorganisms [2, 3].

Traditional herbal medicines are mostly prepared as a combination therapy that has been used since therapeutic was first practiced [18]. Combination therapy is reported as helpful and useful for patients with serious infections caused by drug-resistant pathogens. The purpose of synergistic therapeutic effect is attributed to dose and toxicity reduction and further minimizing the induction of drug resistance [4].

Previous work conducted in our laboratory has already documented the in vitro activities of the extracts of *Senna alata, Ricinus cummunis* and *Lannea barteri* acting alone against some clinically isolated bacterial and fungal species [5]. The goal of this research was to investigate possible extract-extract interaction of the plants as a potential combination therapy against selected clinically isolated pathogenic microorganisms.

## Materials and methods

### Plant materials

The plant material of *S. alata, R. communis* and *L. barteri* were collected from Navrongo (10° 53′ 5.00′′ N, 1° 05′ 25.00′′ W), Ghana, as previously described [6]. The plant was identified and authenticated by a plant taxonomist at the herbarium of Ghana Herbaria, Northern Savanna Biodiversity; Savanna Herbarium. The voucher specimens with numbers of SH 71 (*S. alata*), SH 720 (*R. cummunis*) and SH 790 (*L. Barteri*) were deposited in the herbarium.

### Preparation of plant extracts

Leaf of *S. alata*, leaf of *R. cummunis*, and stem bark of *L. barteri* were thoroughly washed with distilled water, and air dried under shade for two weeks. Each plant sample was pulverized using blender. One hundred grams of each sample was percolated with 1L of (ethanol and water) at room temperature with frequent agitation for 48 h. They were then filtered using Whitman No 42 filter paper, concentrated using rotary evaporator (Heidoph 4001 Efficient) on water bath at 70 °C for the aqueous and 50 °C for the ethanol [6] to obtain a semi-solid and solid residue respectively. A stock solution of 400 mg/mL in dimethyl sulfoxide (DMSO, 99.99%) was prepared from each extract. Extracts were labeled as LBA (*L. barteri* aqueous extract), LBE (*L. barteri* ethanol extract), SAA (*S. alata* aqueous extract), SAE (*S. alata* ethanol extract), RCA (*R. cummunis* aqueous extract) and RCE (*R. cummunis* ethanol extract). Two-fold dilution was used to prepare varying concentrations (200, 100, 50, 25, 12.5, 6.25, 3.13, 1.56 and 0.78 mg/mL) to determine the minimum inhibition concentration (MIC) of each extract alone. For the combination study, the same volume (1:1) of two different extracts (further diluted by two-fold from their respective MICs) were combined. The extracts were stored at ≤ 4 °C until further use.

### Pathogenic microorganisms

Clinical isolates of *Staphylococcus aureus*, *Pseudomonas aeruginosa*, *Escherichia coli*, *Klebsiella pneumoniae and Candida albicans* were obtained from incision-type wounds at the Microbiology Department of the Tamale Teaching Hospital, Tamale Ghana.

### Antimicrobial bioassay

The MIC of the extracts in combination was determined using similar procedure already described by Donkor et al. and Suurbaar et al. [5, 6]. Briefly, overnight cultured bacteria were suspended in 5 mL sterile nutrient broth (NB) and incubated at 37 °C for 2–4 h to achieve a turbidity equivalent to 0.5 McFarland standard (1.0 × 10^8^ cfu/mL). The fungal spores were washed from the surface of agar plates with sterile 0.85% (w/v) saline containing 0.1% Tween 80 (v/v). The spore suspension was adjusted with sterile saline to a concentration of approximately 1.0 × 10^7^ cfu/mL. Sterile NB (2 mL) was inoculated with 0.1 mL of test micororganism in a sterile test tube. The same volume (1:1) of two different extracts (further diluted by two-fold from their respective MICs) were combined in a permutation fashion. The combined extract, 1 mL each, was added to sterile test tubes containing the inocula. The tubes were incubated aerobically at 37 °C for 24 h for bacteria and 48 h for the fungus. The MIC for a combination was calculated as the minimum of the sum of a pair showing no growth. Amoxicillin (2.50 µg/mL) and Fluconazole (1.50 µg/mL) were used as the positive controls for bacteria and fungus respectively.

### Synergy analysis

The extract-extract interactions were analyzed using the fractional inhibition concentration index (FICI) (or ΣFIC) as shown in Eq. 1.

1 $$FICI = \sum FIC =\, \frac{MIC \,of \,extract \,A \,in \,combination}{{MIC \,of \,extract \,A \,alone}} + \frac{MIC \,of \,extract \,B \,in \,combination}{{MIC \,of \,extract \,B \,alone}}$$

The FICI is based on the Loewe additivity zero interaction theory [7–9]. A FICI of ≤ 0.5 indicates synergism, > 0.5 to 1 indicates additive effects, > 1 to ≤ 4 indifference, and > 4 is considered to be antagonism [10, 11].

## Results/discussion

In vitro, extract-extract interactions between the selected medicinal plants on the test clinical microbial isolates were conducted in the current work. Different extracts from the same plant were not considered. The MICs of the extracts acting alone were reported in a previous study conducted in our laboratory [5, 6]. It was observed that MICs ranged between 3.13 and 25.00 mg/mL. In the current work, the MICs of the extracts in combination are shown in Table 1 (and Additional file 1: Table S1). It was observed that the combined extracts gave considerably lower MICs against the entire tested microorganisms than the extracts alone. The MICs of the extracts in combination were species-specific, ranging from 0.97 to 1.17, 0.97 to 4.69, 0.50 to 1.17, 1.17 to 3.12 and 2.34 to 4.69 mg/mL for *E. coli*, *S. aureus, P. aeruginosa*, *K. pneumoniae* and *C. albicans* respectively. The lower MICs could be attributed to the enhanced inhibitory activities of the extracts in a combined form. Synergistic studies between extract-extract combinations of plants are limited in the literature, making a comparison of results from different studies exceptionally challenging. Obuekwe et al. [12] reported that different zones of inhibition were measured using diverse extract combinations and indicated that *E. coli* exhibited a maximum zone concerning the extracts combination of *Bryophyllum pinnatum* and *Ocimum gratissimum* in 30 mg/mL concentration (27.25 ± 1.70 mm). Nonetheless, the utmost zone of inhibition was detected with *S. aureus* in 10 mg/ml concentration regarding ethyl acetate extracts of *Ocimum gratissimum* and *Ficus exasperata* (31.75 ± 3.07 mm) [12]. Related research reports indicated that drug interactions between known antibiotics and plant extracts have potential benefits [4].

**Table 1:** Representative MIC (mg/mL) and FICI of *L. barteri*, *S. alata* and *R. communis* extracts in combination

LBA + SAA	*E. coli*	*S. aureus*	*P. aeruginosa*	*K. pneumoniae*	*C. albicans*
MIC	1.17	3.12	1.56	2.34	3.12
FICI	0.56	0.50	0.50	0.56	0.50
Effect	A	S	S	A	S

LBA + SAE	*E. coli*	*S. aureus*	*P. aeruginosa*	*K. pneumoniae*	*C. albicans*
MIC	1.56	1.95	1.56	3.12	3.12
FICI	0.50	0.47	0.50	0.50	0.50
Effect	S	S	S	S	S

LBA + RCA	*E. coli*	*S. aureus*	*P. aeruginosa*	*K. pneumoniae*	*C. albicans*
MIC	1.56	1.95	1.17	3.12	3.12
FICI	0.50	0.78	0.56	0.50	0.50
Effect	S	A	A	S	S

LBA + RCE	*E. coli*	*S. aureus*	*P. aeruginosa*	*K. pneumoniae*	*C. albicans*
MIC	1.56	4.69	1.56	1.95	2.44
FICI	0.50	0.56	0.50	0.47	0.29
Effect	S	A	S	S	S

*MIC*  Minimum inhibition concentration, *FICI*  Fractional inhibition concentration index, S  Synergy, *A*  Additive; *LBA*  *Lannea barteri* aqueous extract, *LBE*  *Lannea barteri* ethanol extract, *SAA*  *Senna alata* aqueous extract, *SAE*  *Senna alata* ethanol extract, *RCA*  *Ricinus communis* aqueous extract, *RCE*  *Ricinus communis* ethanol extract

The combination of more than one category of drug constituent is regularly used in medicine. A particular strategy does not constantly enrich its particular pharmacological effect; combinations comprising two or more components can afford additive, synergistic, or antagonistic effects [19]. Records show that if treatment using a drug combination gives a better pharmacological effect than monotherapy in clinical trials, it can be concluded that it has a synergistic effect [13]. Kumar et al. [14] reported the synergistic activity of oxytetracycline with methanol extract of *Thespesia populnea*. They observed that the MICs of the combination on 12 different Gram-positive and Gram-negative bacteria were around 62.5–1000 µg/mL.

In the current study, it was observed that only LBA + SAE and SAA + RCE combinations showed a synergy effect on all the test microorganisms. However, the other combinations exhibited a synergy effect on at least one microorganism, though some showed an additive effect. For example, LBA + SAA had a synergy effect on all microorganisms except on *E. coli* which indicated additivity. Furthermore, none of the combinations produced an antagonistic or indifference mechanism (Table 1, Additional file 1: Table S1). According to Caesar and Cech [15], combination therapy is more likely to prevent disease resistance than single pure active constituents. For instance, it is reported that purified artemisinin as monotherapy is more likely to develop resistance in *Plasmodium* parasites than whole-plant *Artemesia annua* extracts or refined fractions [16]. It is on this score that the World Health Organization (WHO) has promoted the use of artemisinin combination therapies (ACTs) in the treatment of *Plasmodium falciparum* malaria to enhance efficacy and reduce resistance. Another renowned antimicrobial agent exhibiting a significant synergistic effect in combination is Co-amoxiclav [20].

In a combined therapy, each bioactive compound exerts a synergistic or an additive effect, thus improving therapeutic efficacy while reducing dose and toxicity. Additionally, an outcome of synergism resulting from combination therapy is to minimize or delay the emergence of resistance by the pathogenic organism to the individual components of the combination [4, 17]. The synergy and additive effects exhibited by the extract-extract interactions in the current work could be attributed to the different phyto-compounds in the individual extracts [5, 6] acting in concert, each introducing a different mechanism to inhibit or kill the microbial growth.

Phytochemical screening showed the presence of most or all of the following: tannins, saponins, polyuronoids, reducing sugars, terpenoids, flavonoids and alkaloids in both aqueous and ethanolic extracts of *S. alata, L. barteri and R. communis.* Alkaloids are reported to have cytotoxicity properties [21]. Phenanthroindolizidine plant alkaloids are reported to inhibit the activity of dihydrofolate reductase, thereby inhibiting nucleic acid synthesis [22]. Additionally, alkaloids, terpenoids, and phenolic compounds are known to inhibit bacterial and fungal infections [23]. Saponins are reported to interfere with cell replication, including cancer cells [24]. Tannins are linked with complexing with proteins through both covalent and non-covalent interactions, and capable of complexing with polysaccharides [25]. Synergy might have resulted from the targeting of multiple pathways, which may include substrates, enzymes, metabolites, ion channels, ribosomes, and signal cascades [26].

## Conclusions

Traditional medicines have a wide-ranging treatment concept, and have the advantages of multiple targets, links, and approaches. The current findings validate the relevance of combining *S. alata, R. communis* and *L. barteri* in treating infections by traditional health practitioners and the need for comprehensive studies in vivo.

## Limitations

This work was carried out on a fixed ratio of 1:1 instead of considering various ratios. This could be a limitation because combination effects may occur over a vast range of concentrations.

## Supplementary Information

**Additional file 1: Table S1.** MICand FICI of *Lannea barteri*, *Senna alata* and *Ricinus communis* extracts in combination.

## Data Availability

The datasets used and/or analyzed during the current study are available from the corresponding author upon reasonable request.

## Abbreviations

A: Additive
*C. albicans*: *Candida albicans*
cfu: Colony forming unit
*E. coli*: *Escherichia coli*
FICI: Fractional inhibition concentration index
*K. pneumoniae*: *Klebsiella pneumoniae*
*L. barteri*: *Lannea barteri*
LBA: *Lannea barteri* aqueous extract
LBE: *Lannea barteri* ethanol extract
MIC: Minimum inhibition concentration
*P. aeruginosa*: *Pseudomonas aeruginosa*
*R. communis*: *Ricinus communis*
RCA: *Ricinus communis* aqueous extract
RCE: *Ricinus communis* ethanol extract
S: Synergy
*S. alata*: *Senna alata*
SAA: *Senna alata* aqueous extract
SAE: *Senna alata* ethanol extract
*S. aureus*: *Staphylococcus aureus*
