# Supplementary material for: Combination therapy: synergism among three plant extracts against selected pathogens
Source: BMC Res Notes. 2023 May 20;16:83. doi: 10.1186/s13104-023-06354-7 (PMC10199525; doi:10.1186/s13104-023-06354-7)
Supplement: Supplementary file 1 — Additional file 1: Table S1. MICand FICI of Lannea barteri, Senna alata and Ricinus communis extracts in combination. [file 13104_2023_6354_MOESM1_ESM.docx]

Table S1: MIC (mg/mL) and FICI of *Lannea barteri*, *Senna alata* and *Ricinus communis* extracts in combination

| LBA + SAA | *E. coli* | *S. aureus* | *P. aeruginosa* | *K. pneumoniae* | *C. albicans* |
| --- | --- | --- | --- | --- | --- |
| MIC | 1.17 | 3.12 | 1.56 | 2.34 | 3.12 |
| FICI | 0.56 | 0.50 | 0.50 | 0.56 | 0.50 |
| Effect | A | S | S | A | S |
|  |  |  |  |  |  |
| LBA + SAE | ***E. coli*** | ***S. aureus*** | ***P. aeruginosa*** | ***K. pneumoniae*** | ***C. albicans*** |
| MIC | 1.56 | 1.95 | 1.56 | 3.12 | 3.12 |
| FICI | 0.50 | 0.47 | 0.50 | 0.50 | 0.50 |
| Effect | S | S | S | S | S |
|  |  |  |  |  |  |
| LBA + RCA | ***E. coli*** | ***S. aureus*** | ***P. aeruginosa*** | ***K. pneumoniae*** | ***C. albicans*** |
| MIC | 1.56 | 1.95 | 1.17 | 3.12 | 3.12 |
| FICI | 0.50 | 0.78 | 0.56 | 0.50 | 0.50 |
| Effect | S | A | A | S | S |
|  |  |  |  |  |  |
| LBA + RCE | ***E. coli*** | ***S. aureus*** | ***P. aeruginosa*** | ***K. pneumoniae*** | ***C. albicans*** |
| MIC | 1.56 | 4.69 | 1.56 | 1.95 | 2.44 |
| FICI | 0.50 | 0.56 | 0.50 | 0.47 | 0.29 |
| Effect | S | A | S | S | S |
|  |  |  |  |  |  |
| LBE + SAA | ***E. coli*** | ***S. aureus*** | ***P. aeruginosa*** | ***K. pneumoniae*** | ***C. albicans*** |
| MIC | 1.17 | 3.12 | 1.56 | 1.56 | 3.12 |
| FICI | 0.56 | 0.50 | 0.50 | 0.50 | 0.50 |
| Effect | A | S | S | S | S |
|  |  |  |  |  |  |
| LBE + SAE | ***E. coli*** | ***S. aureus*** | ***P. aeruginosa*** | ***K. pneumoniae*** | ***C. albicans*** |
| MIC | 1.56 | 3.13 | 1.56 | 2.34 | 3.12 |
| FICI | 0.50 | 0.50 | 0.50 | 0.56 | 0.50 |
| Effect | S | S | S | A | S |
|  |  |  |  |  |  |
| LBE + RCA | ***E. coli*** | ***S. aureus*** | ***P. aeruginosa*** | ***K. pneumoniae*** | ***C. albicans*** |
| MIC | 1.56 | 1.95 | 0.5 | 1.17 | 3.12 |
| FICI | 0.50 | 0.78 | 0.24 | 0.28 | 0.50 |
| Effect | S | A | S | S | S |
|  |  |  |  |  |  |
| LBE + RCE | ***E. coli*** | ***S. aureus*** | ***P. aeruginosa*** | ***K. pneumoniae*** | ***C. albicans*** |
| MIC | 1.56 | 3.12 | 1.56 | 1.56 | 4.69 |
| FICI | 0.50 | 0.50 | 0.50 | 0.50 | 0.56 |
| Effect | S | S | S | S | A |
|  |  |  |  |  |  |
| SAA + RCA | ***E. coli*** | ***S. aureus*** | ***P. aeruginosa*** | ***K. pneumoniae*** | ***C. albicans*** |
| MIC | 0.97 | 0.97 | 1.17 | 1.17 | 3.12 |
| FICI | 0.47 | 0.39 | 0.56 | 0.28 | 0.50 |
| Effect | S | S | A | S | S |
|  |  |  |  |  |  |
| SAA + RCE | ***E. coli*** | ***S. aureus*** | ***P. aeruginosa*** | ***K. pneumoniae*** | ***C. albicans*** |
| MIC | 0.98 | 2.34 | 1.56 | 1.96 | 3.12 |
| FICI | 0.47 | 0.28 | 0.50 | 0.39 | 0.37 |
| Effect | S | S | S | S | S |
|  |  |  |  |  |  |
| SAE + RCA | ***E. coli*** | ***S. aureus*** | ***P. aeruginosa*** | ***K. pneumoniae*** | ***C. albicans*** |
| MIC | 1.56 | 1.95 | 1.17 | 3.12 | 3.12 |
| FICI | 0.50 | 0.78 | 0.56 | 0.50 | 0.50 |
| Effect | S | A | A | S | S |
|  |  |  |  |  |  |
| SAE + RCE | ***E. coli*** | ***S. aureus*** | ***P. aeruginosa*** | ***K. pneumoniae*** | ***C. albicans*** |
| MIC | 1.56 | 2.34 | 1.56 | 2.34 | 2.34 |
| FICI | 0.50 | 0.28 | 0.50 | 0.56 | 0.28 |
| Effect | S | S | S | A | S |

MIC= Minimum inhibition concentration; FICI = Fractional inhibition concentration index; S = Synergy; A = Additive; LBA = *Lannea barteri* aqueous extract; LBE = *Lannea barteri* ethanol extract; SAA = *Senna alata* aqueous extract; SAE = *Senna alata* ethanol extract; RCA = *Ricinus communis* aqueous extract; RCE = *Ricinus communis* ethanol extract
